# Nitrogen Cycling Responses to Mountain Pine Beetle Disturbance in a High Elevation Whitebark Pine Ecosystem

**DOI:** 10.1371/journal.pone.0065004

**Published:** 2013-06-05

**Authors:** Megan P. Keville, Sasha C. Reed, Cory C. Cleveland

**Affiliations:** 1 Department of Ecosystem and Conservation Sciences, University of Montana, Missoula, Montana, United States of America; 2 U.S. Geological Survey, Southwest Biological Science Center, Moab, Utah, United States of America; DOE Pacific Northwest National Laboratory, United States of America

## Abstract

Ecological disturbances can significantly affect biogeochemical cycles in terrestrial ecosystems, but the biogeochemical consequences of the extensive mountain pine beetle outbreak in high elevation whitebark pine (WbP) (*Pinus albicaulis*) ecosystems of western North America have not been previously investigated. Mountain pine beetle attack has driven widespread WbP mortality, which could drive shifts in both the pools and fluxes of nitrogen (N) within these ecosystems. Because N availability can limit forest regrowth, understanding how beetle-induced mortality affects N cycling in WbP stands may be critical to understanding the trajectory of ecosystem recovery. Thus, we measured above- and belowground N pools and fluxes for trees representing three different times since beetle attack, including unattacked trees. Litterfall N inputs were more than ten times higher under recently attacked trees compared to unattacked trees. Soil inorganic N concentrations also increased following beetle attack, potentially driven by a more than two-fold increase in ammonium (NH_4_^+^) concentrations in the surface soil organic horizon. However, there were no significant differences in mineral soil inorganic N or soil microbial biomass N concentrations between attacked and unattacked trees, implying that short-term changes in N cycling in response to the initial stages of WbP attack were restricted to the organic horizon. Our results suggest that while mountain pine beetle attack drives a pulse of N from the canopy to the forest floor, changes in litterfall quality and quantity do not have profound effects on soil biogeochemical cycling, at least in the short-term. However, continuous observation of these important ecosystems will be crucial to determining the long-term biogeochemical effects of mountain pine beetle outbreaks.

## Introduction

Whitebark pine (*Pinus albicaulis*; hereafter referred to as WbP) – a coniferous tree species common in western North America – is considered a keystone species in subalpine ecosystems [1], [2]. Recently, WbP has been the subject of both considerable research and media attention because this important species is facing a number of threats to its existence. WbP has already experienced significant population declines across its distribution [3], and if widespread mortality of WbP continues, the species is in danger of becoming functionally extinct [4]. Among the most serious threats to WbP is the current mountain pine beetle (*Dendroctonus ponderosae,* MPB) outbreak, unprecedented in terms of its extent, particularly in WbP ecosystems [5]. Unfortunately, it is still unclear how this disturbance may affect many ecosystem processes – including nutrient cycling – in WbP dominated ecosystems. Nevertheless, understanding the potential effects on biogeochemistry are important, because nutrient cycling could play a critical role in determining the trajectory of recovery and regeneration of these ecologically important ecosystems following disturbance [6].

Changes in biogeochemical cycling following ecological disturbances have been documented in a wide range of ecosystems [7]–[10], and nitrogen (N) cycling, in particular, has been shown to change dramatically and rapidly following disturbance [11]–[13]. Such changes can be ecologically significant for several reasons. For example, N often limits ecosystem processes, particularly in high elevation temperate forests [14]. Thus changes in N pools and fluxes may impact a range of ecosystem characteristics [15]–[17]. Because N is relatively mobile and can be easily lost from ecosystems (e.g., via leaching; [18]–[20]), disturbances that mobilize N can further deplete the availability of an already potentially limiting nutrient. In general, disturbance-induced forest mortality can initiate rapid increases in forest floor N due to both increases in dead biomass inputs and reduced plant N demand and uptake. This N can then be quickly lost, as large, episodic inputs of organic N are typically followed by increases in N mineralization and subsequent N losses through NO_3_^−^ leaching and denitrification [10]. However, while some studies have observed significant long-term shifts in N pools and fluxes following disturbance [18], [21], [22], other studies have shown that effects can be modest and short-lived [23], [24], [25]. This suggests that the magnitude and longevity of biogeochemical responses to disturbances such as insect-induced plant mortality are dependent upon characteristics of the affected ecosystem and/or the outbreak itself.

The mountain pine beetle is a phloem-feeding insect that typically undergoes outbreaks in pine forests of the western U.S. and Canada over 30–40 year cycles, and some of the largest WbP populations in the Greater Yellowstone Ecosystem experienced a 320-fold increase in the number of MPB-infested trees between 1999–2007 [4], [26]. During an attack, beetles deposit their eggs in the phloem of a host pine tree where the larvae develop and feed on the phloem [27]. As the larvae feed, they effectively girdle the tree, often leading to mortality within two weeks of a successful attack [5]. For approximately five years following attack, infested stands undergo a number of changes that have the potential to influence biogeochemical cycling. For example, shortly following beetle attack, nutrient and water uptake by host trees ceases, potentially altering soil moisture and soil nutrient pools [13], [28], [29]. Within two years of attack, trees enter the “red” stage of beetle infestation, when needles typically turn red and begin falling to the ground. In other host tree species where litterfall nutrient content has been analyzed, attacked tree litterfall typically has higher N concentrations than normally senescing litterfall, because the attacked trees do not resorb nutrients from their needles before they fall [13], [20], [30]. This large, relatively rapid pulse of needlefall during the red stage provides substantial inputs of carbon (C) and N to the forest floor [29] several years after attack. Five years after attack, trees have typically lost all their needles to the forest floor and reach the “gray” stage.

We took advantage of an ongoing “natural experiment” initiated by the current beetle outbreak to investigate the effects of tree mortality as a result of beetle attack on N cycling in a WbP pine ecosystem. We measured a suite of N cycling metrics under WbP trees occupying three different stages of beetle attack in the Pioneer Mountains of southwestern Montana: ‘green’ (unattacked) stage, ‘red’ stage, and ‘gray’ stage. Given observed changes in N cycling following beetle disturbance in other types of forest ecosystems [13], [29], [30], we began with several hypotheses. First, we hypothesized that litterfall mass would be significantly higher under red stage trees compared to green and gray stage trees, and that litterfall C:N ratios would be lower under infested trees (reflecting minimal foliar nutrient resorption in attacked trees; [29], [30]). Second, we hypothesized that soil inorganic (NH_4_^+^+NO_3_^−^) and organic N concentrations would be higher under red and gray stage trees compared to green trees. An increase in available soil N could have many possible fates, including microbial immobilization, uptake by understory plants, and/or conversion of NH_4_^+^ to NO_3_^−^ with the potential for loss from the ecosystem through leaching [18], [21], [22], [28]. Thus, in response to increased N inputs under attacked trees, we hypothesized increases in soil N mineralization and nitrification rates and understory plant foliar N concentrations.

## Methods

### 2.1. Study Site

**Figure 1:**
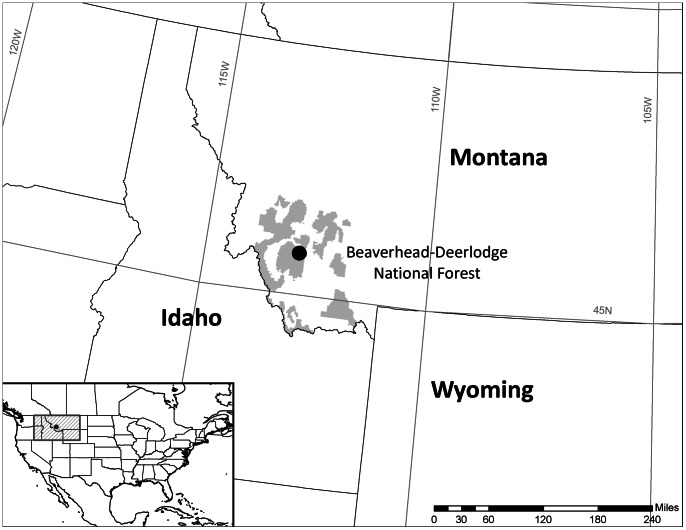
Map of study site. Location of study site, Vipond Park (filled circle) in the Beaverhead-Deer Lodge National Forest (gray shading) in southwestern Montana.

The study was conducted at Vipond Park (45.697° N, −112.910° W) in the Pioneer Mountains in the Beaverhead-Deerlodge National Forest of southwestern Montana, USA (Fig. 1). All necessary field permits were obtained from the Beaverhead-Deer Lodge National Forest. The site elevation is 2500 m and average temperatures in the region range from −9°C in January to 13°C in July (SNOTEL site 656, 2530 m, 1979–2009 average). Mean annual precipitation is approximately 770 mm falling mostly as snow (SNOTEL site 656, 2530 m 1979–2009 average), and snow covers the ground for approximately 8 months per year. Soils in the area consist of Typic calcicryepts (Inceptisols) and Eutic haplocryalfs (Alfisols) derived from limestone colluvium parent materials (USDA Natural Resources Conservation Service, Web Soil Survey; http://websoilsurvey.nrcs.usda.gov/app/HomePage.htm). The site contains an open canopy forest with whitebark pine (*Pinus albicaulis)* as the dominant canopy tree species co-occurring with occasional lodgepole (*Pinus contorta)* and limber pine (*Pinus flexilis)*. The understory is sparse, consisting primarily of perennial grasses and forbs. The current mountain pine beetle outbreak at Vipond Park was first observed in 2005 and, at the time of the study, had progressed to the point where over 70% of the WbP trees are at some stage of beetle infestation (Diana Six, personal communication).

### 2.2. Sampling Design

WbP at the study site does not grow in a stand per se, but in “clumps” or “patches” [31] consisting of several individuals. Thus, to assess the influence of WbP mortality resulting from beetle kill on N cycling, we established ten 4×4 m (16 m^2^) plots centering around individual “focal trees” [32]. This sampling design allowed for the isolation of tree-level effects of beetle infestation at a site that is relatively heterogeneous (as most WbP stands are) in terms of both canopy cover and beetle attack. Three common stages of beetle infestation were investigated in this study: 1) ‘*green’*, uninfested WbP; 2) ‘*red’*, recently infested WbP (within 2 years) with needles that had turned red but had not fallen; and 3) ‘*gray’*, WbP trees infested more than two years ago and that lacked needles. These three categories reflect approximate times since tree death and depict significant changes in terms of potential N fluxes. Ten WbP pine individuals at each of the three infestation stages (30 trees total) were used as focal trees. Focal tree locations were selected based on where patches of trees of a particular beetle infestation stage occurred (i.e., focal trees were adjacent to trees of the same infestation stage/mortality state). Each focal tree was at least 30 m from every other focal tree. In addition to beetle infestation stage, focal trees were chosen based on a >15 cm minimum diameter at breast height (DBH) threshold, as this is considered to be the cutoff size that is conducive to beetle attack. In addition, all focal trees were relatively similar in size, ranging from 15–40 cm DBH.

At each focal tree, soil samples were collected at four different points between the bole and the crown drip line of the tree, in each of four cardinal directions (90° apart) starting 0.5 m from the base of the tree and rotating outward in 0.5 m increments. Soil organic horizon samples were collected to a depth of approximately 5 cm at each sampling point. Mineral soil samples were collected directly below the organic horizon cores to 15 cm depth using an eight cm diameter hand corer. In cases where rocks obstructed the mineral cores, samples were taken adjacent to the organic sampling point after removing the organic material from the mineral soil surface. During each sampling event, soil samples from each of the two depths were composited by tree (i.e., for each three there was an organic and mineral soil horizon sample), placed in coolers and transported to the laboratory at the University of Montana for analysis. Within 48 h, soil samples were sieved (to 2 mm) and subsampled for physical and chemical analyses including gravimetric moisture content, inorganic N concentrations and microbial biomass analysis (see below), and the remaining soil was air-dried for pH and dried at 105°C for total C and N analyses. Soil pH was determined at 2∶1 (water: soil) (Beckman Instruments, Fullerton, CA).

Focal tree, soil, and understory plant sampling were done in three phases. First, WbP focal tree foliage, litterfall and standing litter assessments were performed in July 2010. Soil samples were collected in September and October 2010; in September, soil inorganic N concentrations and potential N mineralization rates were assayed, and in October soil microbial biomass and total soil C and N concentrations were assessed. Understory cover and foliar chemistry was determined in July of 2011.

### 2.3. Foliage and Litter Quantity and Quality

One 0.25 m^2^ litter trap was placed 0.5 m from the bole of each focal tree in July 2010. Litter was collected after one month to obtain an index of relative litterfall among beetle infestation stages and to perform litter C and nutrient analyses. Following collection, litter was dried at 70°C for 48 hours and subsequently weighed. Oven-dried subsamples from each litter trap were ground with a Wiley Mill (20-mesh screen), weighed into tin capsules (approximately 4 mg sample), and combusted on a CHNS-O elemental analyzer (CE Instruments EA 1110, Thermo Fisher Scientific, Waltham, MA, USA) to determine total litter C and N concentrations (University of Montana Environmental Geochemistry Laboratory, University of Montana, Missoula, MT).

Canopy foliage was collected in July 2010 from green and red stage trees. Samples were taken approximately 2.5 m from the ground at various points around each tree. All green and red foliage was collected near the branch tips in order to maintain consistency with respect to needle cohort. Foliage was analyzed for total C and N concentration using the above protocols. Standing litter was sampled from below each focal tree in July 2010. A 0.25 m^2^ sampling quadrat was placed 0.5 m away from each tree and all intact standing litter in the quadrat was collected down to the organic horizon, dried at 70°C for 48 hours and weighed. Subsamples were ground, weighed into tin capsules (approximately 4 mg sample), and combusted on a CHNS-O elemental analyzer (CE Instruments) for total standing litter C and N.

### 2.4. Understory Vegetation and Foliar N

Understory vegetation under each focal tree was measured in July 2011. Two 0.25 m^2^ quadrats were placed 0.5 m from the bole of each tree oriented toward the north and south. Percent ground cover for all understory species in each quadrat was assessed using ocular estimation according to plant functional group (grass, forb, sedge, shrub, seedling). Foliar samples from two common understory species, *Viola praemorsa* (Canary violet) and *Oxytropis sericea* (Silky locoweed*)*, were collected from the quadrats and composited by focal tree and species. Samples were oven-dried at 70°C for 48 hours, ground as described above, weighed into tin capsules (approximately 4 mg sample), and combusted on a Carlo Erba elemental analyzer for total C and N concentration.

### 2.5. Soil N Pools

Within 24 hours of collection, soil inorganic N (ammonium [NH_4_^+^] and nitrate [NO_3_^−^]) was extracted from the September 2010 soil samples. Ten grams of each soil sample were extracted in 2 M KCl for 18 hours, vacuum filtered through 11 µm Whatman (Grade 1) filter paper, and stored at 4°C until analysis. Extracts were analyzed colorimetrically for inorganic N (NH_4_^+^ and NO_3_^−^) using a Synergy 2 Microplate Reader (BioTek, USA) after Weatherburn [33] and Doane and Horwath [34], for NH_4_^+^ and NO_3_^−^, respectively.

Soil microbial biomass C and N concentrations were analyzed using the chloroform fumigation-extraction method [35]. Briefly, ten grams each of chloroform-fumigated and unfumigated samples from the October 2010 collection (organic and mineral samples composited) were extracted with 0.5 M K_2_SO_4_ for one hour and vacuum-filtered through 11 µm Whatman (Grade 1) filter paper. Organic C and total N in extracts were analyzed using a Shimadzu TOC-V CPN/TNM-1 analyzer (Shimadzu, Inc, Kyoto, Japan). Microbial biomass C was determined as the difference between extractable organic C in fumigated and unfumigated samples using a proportionality constant (K*c*) of 0.45 [36]. Microbial biomass N was determined the same way except using a correction factor (K*n*) of 0.54 [35]. Finally, composited organic and mineral soil samples from the October 2010 soil collection were ground, weighed into tin capsules (approximately 7 mg samples), and combusted on a CHNS-O elemental analyzer for total soil C and N determination.

### 2.6. Soil N Transformations and Fluxes

#### 2.6.1. N mineralization/net nitrification rates

We conducted a 28-day laboratory incubation of soil samples collected in September 2010 (organic and mineral horizons kept separate) to assess net N mineralization rates. Ten grams of field moist, sieved soil were mixed and weighed into plastic vials, covered with perforated plastic wrap, and incubated in the dark at room temperature (22°C) for 28 days. Vials were reweighed weekly and deionized water was added to maintain field moisture of each sample. After 28 days, samples were analyzed for NH_4_^+^ and NO_3_^−^ as described above (after Weatherburn [33] and Doane and Horwath [34], respectively.) Net N mineralization rates were calculated by subtracting the pre-incubation September 2010 inorganic N values from the final (post-incubation) values, and dividing by the number of incubation days (i.e., 28). Net nitrification rates were obtained in the same way, using NO_3_^−^ concentrations instead of inorganic N (NH_4_^+^+NO_3_^−^) concentrations.

#### 2.6.2. Soil soluble N fluxes

Soil N fluxes were assessed using ion-exchange resin capsules (Unibest, Inc, Bozeman, MT, USA). Capsules were deployed in the field twice, first capsules remained in soil from July – October 2010, and second capsules remained in soil from October – July 2011, following snowmelt. Resin capsules were inserted in soil beneath each focal tree to 10–15 cm depth by carefully creating a slit in the soil with a hand trowel, inserting the capsule, and carefully removing the blade to minimize disturbance. Once the capsules were collected from the field, resin-exchanged inorganic N (NH_4_^+^+NO_3_^−^) concentrations were determined following extraction in 2 M KCl and colorimetric analysis.

### 2.7. Statistical Analyses

All statistical analyses were conducted using SPSS v. 19 (IBM SPSS, Inc., Chicago, IL, USA). Analysis of variance (ANOVA) was used to determine significant differences between the three beetle infestation stages for litterfall mass, standing litter mass, litter C and N concentrations, soil C and N pools and fluxes, and understory vegetation cover and nutrient concentrations. The assumptions of normality and homogeneity of variances were met for all data. Repeated measures ANOVA was used to test for the effects of both time and beetle infestation stage on soil moisture. The normality, homogeneity of variance, and sphericity assumptions were all satisfied. For *post-hoc* analyses, Tukey’s HSD test was used to determine differences between stages when ANOVAs were significant (α = 0.05).

## Results

### 3.1. Soil Abiotic Characteristics

Soil pH did not vary significantly with beetle infestation stage (Table 1), but soil moisture was higher under both the red and gray stage trees (P = 0.03 and P = 0.04, respectively) compared with soil moisture soil under the green stage trees. The repeated measures ANOVA indicated that sampling time was a significant factor in explaining differences in soil moisture over the course of the growing season (P<0.001; Table 1). There was not a significant time × infestation stage interaction. However, infestation stage alone explained a significant proportion of the variation in soil moisture among treatments (Table 1).

**Table 1:** Mean (±1 SE) soil characteristics under green, red and gray stage whitebark pine trees at Vipond Park in southwestern Montana.

	Infestation Stage		
	Green	Red	Gray
Soil moisture (%)			
Organic horizon			
* July 2010*	37.43 (7.71) a *	49.32 (11.48) b *	45.82 (7.58) b *
* August 2010*	22.77 (10.39) a **	34.30 (13.73) b **	32.80 (14.79) b **
* September 2010*	37.44 (9.22) a *	44.93 (9.52) b *	47.93 (12.80) b *
Mineral horizon			
* July 2010*	26.77 (7.05) a *	24.29 (3.03) a *	27.34 (4.42) a *
* August 2010*	8.31 (6.67) a **	12.66 (4.85) ab **	15.05 (6.51) b **
* September 2010*	19.93 (3.45) a ***	24.22 (2.55) b *	24.38 (4.41) b *
Bulk soil pH (0–15 cm)	6.17 (0.22) a	6.44 (0.21) a	6.30 (0.19) a
Bulk soil C (%)	7.43 (2.66) a	8.48 (3.23) a	9.72 (4.73) a
Bulk Soil N (%)	0.34 (0.09) a	0.38 (0.14) a	0.41 (0.18) a
Bulk soil C:N	21.25 (1.24) a	22.51 (1.01) a	23.32 (1.35) a

Green stage represents unattacked trees, red stage, trees attacked within the last five years, and gray stage, trees attacked>five years ago. Lower case letters denote significant differences among stages (rows) and number of stars denotes significant differences among sampling dates (columns). Letters and stars denote significant differences at α = 0.05.

### 3.2. Litterfall: Relative Rates and Chemistry

In July 2010, litterfall inputs were more than an order of magnitude higher under the red stage trees (278.38±44.08 g/m^2^) than under either green stage or gray trees (11±5 g/m^2^ and 77±21 g/m^2^ respectively; P<0.001 for both). However, July 2010 litterfall inputs did not vary significantly between the gray and green stages (Table 2). Total litterfall N concentrations were significantly higher in gray and red stage litterfall (1.45±0.05% and 1.16±0.07%, respectively) compared to green stage litterfall (1.06±0.04%) (P = 0.001; P<0.001, respectively). This was reflected in the significantly higher litterfall N pools under the red and gray stages compared to the green stage (P<0.001 for both) (Table 2). Litterfall C:N ratios varied between stages, with the red and gray stages having significantly lower C:N ratios than the green stage needles (P = 0.046; P<0.001 respectively; Table 2). Foliar C:N ratios varied as well, with the green stage trees having approximately double the foliar C:N (109.88±2.35) of the red stage trees (55.66±2.91; P<0.001). There were no significant differences in standing litter mass among infestation stages, although the red and silver stages tended to be higher than the green stage (Table 2). Similarly, standing litter N and C:N did not vary among infestation stages.

**Table 2:** Mean (±1 SE) N input characteristics under green, red and gray stage whitebark pine trees at Vipond Park in southwestern Montana.

	Infestation Stage		
	Green	Red	Gray
Litterfall Mass (g/m^2^)	11.05 (5.23) **a**	278.38 (44.08) **b**	77.48 (21.31) **a**
Litterfall N (%)	1.06 (0.12) **a**	1.16 (0.21) **b**	1.45 (0.15) **b**
Litterfall C:N	48.10 (5.38) **a**	41.25 (7.97) **b**	34.67 (4.34) **b**
Litter Mass (g/m^2^)	1938.99 (261.79) a	2994.08 (451.98) a	2655.96 (517.99) a
Litter N (%)	1.48 (0.31) a	1.56 (0.30) a	1.63 (0.20) a
Litter C:N	31.66 (5.69) a	29.25 (5.98) a	27.17 (2.45) a
Organic soil inorganic N (µg N/g soil)	14.96 (6.97) **a**	33.60 (15.76) **b**	21.19 (11.58) **ab**
Mineral soil inorganic N (µg N/g soil)	4.86 (2.96) a	4.73 (2.93) a	6.32 (3.89) a

‘Green’ stage represents unattacked trees, ‘red’ stage, trees attacked within the last five years, and ‘gray’ stage, trees attacked>five years ago. Letters denote significant differences between stages at α = 0.05.

### 3.3. Soil N Pools

Soil inorganic N concentrations (NH_4_^+^+NO_3_^−^) were higher (P<0.001) in the organic horizon compared to the mineral horizon for all stages. Across infestation stages, the organic horizon under red stage trees had higher extractable inorganic N concentrations than the organic horizon of either the green or gray stage trees (P = 0.005, P = 0.071) (Table 2). These differences in inorganic N were driven by changes in NH_4_^+^ across infestation stages. In the mineral horizon, extractable inorganic N did not vary across infestation stages (Table 2) and soil NO_3_^−^ concentrations were very low (i.e., near detection limits) and highly variable for all mineral soils measured. There were no differences in NO_3_^−^ levels across infestation stages in either soil horizon, and there were no significant differences in either microbial biomass N concentrations or total soil N concentrations across infestation stages in either the organic or mineral soil horizons (Tables 1 and 3). However, soil microbial biomass C:N ratios were significantly lower under the red stage trees compared to the gray stage trees (P = 0.01; Table 3).

**Table 3:** Mean (±1 SE) N cycling characteristics under green, red and gray stage whitebark pine trees at Vipond park in southwestern Montana.

	Infestation Stage		
	Green	Red	Gray
N mineralization (µg N/g soil/day)			
Organic horizon	1.51 (0.19) a	1.53 (0.44) a	1.65 (0.36) a
Mineral horizon	0.61 (0.21) a	0.63 (0.19) a	1.05 (0.33) a
Microbial biomass C:N	8.53 (0.40) **ab**	7.65 (0.16) **a**	8.98 (0.30) **b**
Resin Capsule Flux (µg N/g soil)	0.46 (0.17) a	1.13 (0.49) a	0.45 (0.22) a
Understory foliar C:N			
* Silky Locoweed*	8.87 (0.28) a	8.57 (0.17) a	8.35 (0.22) a
* Canary Violet*	11.14 (0.24) a	11.87 (0.83) a	11.27 (0.34) a

Green stage represents unattacked trees, red stage, trees attacked within the last five years, and gray stage, trees attacked>five years ago. Letters denote significant differences between stages. Letters denote significant differences at α = 0.05.

### 3.4. Soil N Cycling

Net soil N mineralization rates were higher in both organic and mineral soils under gray trees, followed by red and then green trees (Table 3), but the differences among stages were not significant. Soil inorganic N fluxes as measured with resin capsules did not vary across stages of beetle infestation (Table 2).

### 3.5. Understory Plant Responses

Understory ground cover did not significantly vary across the three stages of beetle infestation, although grasses and forbs displayed an increasing trend moving from the green to red and gray stages (data not shown). Neither of the two understory forb species (*Viola praemorsa* and *Oxytropis sericea*) exhibited significantly different C:N values across the three stages of beetle infestation (Table 3).

## Discussion

The widespread, unprecedented WbP mortality occurring as a result of the current MPB outbreak has profoundly affected WbP ecosystems, and some research has even suggested that WbP may become “functionally extinct” as a result of the current disturbance [4]. However, what this massive mortality event means for WbP ecosystem biogeochemical cycling remains unknown, and to our knowledge this study is the first to assess how beetle kill alters N cycling at the individual tree level in these high elevation ecosystems. We recognize that our sampling design is effectively a chronosequence, which limits the strength with which we can assert direct causality to the disturbance in the absence of any pre-disturbance data [37]. However, there is no evidence or previous research indicating that factors other than the mountain pine beetle outbreak would have influenced N-cycling changes in our study. We hypothesized that this disturbance would lead to short-term changes in ecosystem N pools and fluxes underneath beetle-infested trees. However, despite some modest changes in soil N cycling in response to the MPB outbreak, overall our results indicate that short-term changes to the N cycle were relatively subtle. This suggests that while the current MPB outbreak may drive large changes in other WbP ecosystem characteristics and ecosystem processes (e.g., succession or disturbance dynamics [4]), changes to the N cycle are relatively small in the short-term, at least compared to the profound effect of the disturbance on other ecosystem characteristics.

Soil moisture varied with beetle infestation stage, such that the red stage trees had the highest soil moisture. There are several possible explanations for this, including changes in transpiration [13], [29], [38], [39], and/or changes in evaporation under green, red or gray trees. For example, increased radiation due to complete loss of canopy cover could promote evaporation, perhaps explaining (at least in part) why soil moisture under gray stage trees was lower than soil moisture under red stage trees. In ponderosa pine stands, Morehouse et al. [30] measured significantly higher photosynthetically active radiation (PAR) reaching the forest floor under beetle-killed trees compared to unattacked trees. Reduced canopy cover of the gray stage trees may partially counteract increases in soil moisture stemming from reduced transpiration. Such changes in soil moisture can have important implications for a number of microbial processes, including N mineralization, respiration and decomposition [40], [41].

Not surprisingly, litterfall inputs were significantly higher under red stage trees compared to green and gray stage trees, reflecting the pulse of litterfall often observed soon after tree mortality following beetle attack. However, increased litterfall (as seen in July) did not translate into differences in standing litter biomass under infested trees. Griffin [13] observed similar patterns in MPB-attacked lodgepole pine stands and attributed this to decomposition breaking down the gradual (3–4 year) influx of litter at a pace that did not allow buildup of a litter layer. Alternatively, the canopy cover patchiness of WbP, as well as the movement of litter during snowmelt or wind and rain events, may contribute to the high variability of standing litter mass, thereby masking detectable shifts among infestation stages.

Litterfall nutrient chemistry varied with infestation stage. Litterfall from gray stage trees had significantly lower C:N ratios than green stage trees, largely driven by higher N concentrations in the needles falling from the infested trees. This result matches the findings of studies conducted in both MPB-attacked lodgepole and ponderosa pine ecosystems [13], [29], [30]. Differences in litterfall nutrient content following MPB attack most likely reflect limited N resorption prior to senescence [30], [42]. However, while we expected these changes in litterfall quantity and quality to translate to subsequent shifts in N cycling as they reached the forest floor and became available for microbial processing and decomposition [12], [20], the shifts we observed for WbP were relatively subtle.

Once increased litterfall N inputs reach the forest floor, the N has multiple possible fates, one of which is uptake by surviving understory vegetation. This could manifest itself through increases in understory net primary productivity, or increases in foliar N concentration of understory plants after the disturbance [43], [44], or both. For example, in lodgepole pine stands attacked by MPB, Griffin et al. [13] observed higher foliar N concentrations in the understory sedge, *Carex geyerii*, in beetle-infested stands compared to uninfested stands, but did not measure higher sedge productivity in infested stands. Other studies of post-beetle stands have observed increases in understory growth in a number of forest types [43], [44], [45]. Increases in available N after disturbance may be responsible for some of this enhanced growth, but other abiotic factors such as increased light and moisture are also likely contributors, particularly in the longer term. We observed no significant differences across infestation stage in the foliar N or C:N ratio of two common understory species (*Viola praemorsa* and *Oxytropis sericea*). There was also no measurable increase in understory ground cover under beetle-attacked trees. Taken together, these data suggest that biogeochemical cycles in different forests may respond differently to attack.

As hypothesized, the highest soil inorganic N values were observed in the organic horizon under red stage trees, and the differences in inorganic N concentrations observed were due to shifts in soil NH_4_^+^ concentrations, not to changes in NO_3_^−^ concentrations. The increase in soil inorganic N under red stage trees could be explained by one or a combination of factors. For example, higher N in litterfall, reduced plant uptake [10], higher microbial N mineralization [7], [38], [30], lower microbial immobilization of N, or transport of N from the N-enriched litter layer [20] could all contribute to the increase in soil inorganic N in the organic horizon in red stage trees. Other studies have reported similar results, with infested stands having significantly more extractable soil NH_4_^+^ than uninfested stands [38], [12], [29]. In beetle-infested vs. uninfested ponderosa pine stands, however, there were no significant differences measured in soil inorganic N pools [30]. In our study, inorganic N concentrations were significantly different and correlated with lower soil microbial C:N ratios. Thus, while the N cycling response is not dramatic and was not observable in our assessment of understory plants, there is the potential for beetle mortality to affect soil N cycling in ways that could feedback to influence soil N availability and loss.

In contrast to the differences in inorganic N in the organic (surface) soil horizons, inorganic N concentrations in the mineral soil horizons did not vary among infestation stages. This may suggest that sufficient time has not elapsed for the increased inputs of N to be transported into the lower mineral horizons. Leaching of inorganic N from the organic horizon and litter layers requires time and movement of water through the soil profile [46]. Alternatively, the increased inorganic N in the organic horizon may be immobilized by microbes, lost via denitrification, or taken up by plants before it can move into the mineral horizon.

While we predicted that the relatively low C:N ratios in litter from beetle-infested trees would drive increases in soil N pools, not all forms of inorganic N shifted following beetle attack. Soil NO_3_^−^ concentrations in both the organic and mineral horizons were consistently very low, highly variable and did not differ among beetle infestation stages. Low soil NO_3_^−^ levels are often measured in high elevation, N-limited ecosystems, as available N is often rapidly taken up by plants or immobilized by microbes [47]. Increases in soil NO_3_^−^ may also have been lost from the ecosystem quickly through leaching during rain events or snowmelt [46]. In other disturbed systems, large increases in soil NO_3_^−^ have been observed, but usually involved nearly 100% vegetation mortality, or took place in systems with substantial N deposition [17], [19]. For example, Orwig [48] measured soil NO_3_^−^ in hemlock stands that had been infested by the insect defoliator, hemlock woody adelgid, and observed significantly higher levels in attacked vs. unattacked stands. These forests were nearly pure hemlock and experienced almost complete mortality as the outbreak moved through the system. Soil texture and the timing of snowmelt or rain events also influence the magnitude of N losses from an ecosystem [6]. Ecosystem characteristics such as climate and soil texture, as well as the extent of disturbance-induced mortality interact to determine how mobilized N behaves following the disturbance, particularly with respect to N losses.

Microbial biomass C:N ratios were lower under red stage trees compared to gray stage trees, but surprisingly red stage trees did not differ from green stage trees. Microbial immobilization of N is one pathway mineralized N may take after a disturbance, particularly if N is limiting [49]. In such cases, lower microbial biomass C:N should reflect this, indicating uptake of N. Potential net N mineralization rates in incubated soils did not vary across beetle infestation stage and this result is not consistent with other insect outbreak studies, which found significantly higher net N mineralization potential in soil from attacked stands [12], [30], [38]. Our results appear to argue against increased microbial N mineralization as the driver behind the observed increase in soil inorganic N in the organic soil horizon. One possibility is that reduced plant uptake resulted in the increase in inorganic N rather than increased N mineralization. Alternatively, the timing of sampling may have missed an initial pulse of mineralization following tree mortality. High elevation WbP ecosystems may undergo the majority of their microbial processing of organic matter and decomposition during spring snowmelt, a time at which our study site is inaccessible.

Inorganic N fluxes through the soil profile (as measured with resin capsules) also did not vary with beetle infestation stage in our site. Nitrogen losses are one of the most commonly measured biogeochemical variables in disturbance studies, and increased NO_3_^−^ levels have been observed in a number of insect defoliation studies (often measured in streams draining the disturbed ecosystem; [7], [18], [21], [22]). For example, a spruce bark beetle outbreak in Germany resulted in elevated N concentrations 40 cm below the soil surface for five years following the outbreak [28]. The fact that no measurable changes to N fluxes were observed in this WbP ecosystem is not entirely surprising considering the very low NO_3_^−^ levels measured in the soil profile.

Overall, the results of this study suggest that, from an N cycling perspective, WbP ecosystems experience relatively subtle changes in the years immediately following beetle attack. While N inputs under focal trees changed significantly following tree mortality, they were accompanied by few shifts in internal cycling and pools of N, at least for the variables we measured. In addition, the internal N cycling characteristics that did change with beetle infestation stage varied in the upper, organic soil horizon, but did not appear in the mineral soil horizon, and there was no evidence suggesting significant shifts in N losses from the system. We would expect gaseous N losses to be very low at this site given the coarse texture of the soil and the dry environment [50]. This does not mean that the differences we did observe are not important, as N could strongly limit ecosystem processes in the system, but overall we did not observe the large N cycling responses we expected based on the dramatic mortality and our focal tree sampling design. Given the lack of strong responses at the level of individual trees, we would also predict that the stand-to-ecosystem scale responses would be similarly subtle, but future research is necessary to test the effects of widespread WbP mortality on N cycling at larger scales. However, it is worth noting that this study indicates that WbP ecosystems react differently to MPB attack compared to studies from lower elevation ecosystems [30], [39], which displayed either stronger or more rapid biogeochemical responses to bark beetle attack.

Considering the extent of WbP mortality at the site, the lack of many significant biogeochemical responses to the increased litter C and N inputs was unexpected. The characteristics of WbP ecosystems, however, provide some insight into potential lags in response time to the disturbance, which may ultimately allow regeneration to occur before any longer-term nutrient shifts occur. For example, the very short growing season and harsh climatic conditions that exist in WbP ecosystems most likely cause many microbial processes to progress more slowly than in other ecosystems. From a biogeochemical perspective, climatic constraints on N cycling could have some benefit. Following this large-scale, high mortality disturbance, major shifts in C and N do not appear to occur over the timeline of a few years, but instead may play out over longer timescales that ultimately do not lead to dramatic N losses. Longer-term monitoring at a number of sites is required to determine whether biogeochemical changes are indeed more subtle in WbP ecosystems, or whether they merely take longer to manifest themselves. Thus far the data suggest that, from an N cycling perspective, the prospects for the future regeneration of WbP may be relatively promising.
